# Non-treatment of children with community health worker-diagnosed fast-breathing pneumonia in rural Malawi: exploratory subanalysis of a prospective cohort study

**DOI:** 10.1136/bmjopen-2016-011636

**Published:** 2016-11-16

**Authors:** Carina King, Tim Colbourn, Limangeni Mankhambo, James Beard, Debbie C Hay Burgess, Anthony Costello, Rasa Izadnegahdar, Norman Lufesi, Charles Mwansambo, Bejoy Nambiar, Eric S Johnson, Robert W Platt, David Mukanga, Eric D McCollum

**Affiliations:** 1 Institute for Global Health, University College London, London, UK; 2 Parent and Child Health Initiative, Lilongwe, Malawi; 3 Kimataifa Diagnostics & Devices Consulting LLC, Seattle, Washington, USA; 4 Bill & Melinda Gates Foundation, Seattle, Washington, USA; 5 Acute Respiratory Infection Unit, Ministry of Health, Lilongwe, Malawi; 6 Ministry of Health, Lilongwe, Malawi; 7 Center for Health Research, Kaiser Permanente Northwest, Portland, Oregon, USA; 8 McGill University, Montreal, Quebec, Canada; 9 Science and Health Impact Group (SHI), Kampala, Uganda; 10 Division of Pulmonology, Department of Pediatrics, Johns Hopkins School of Medicine, Baltimore, Maryland, USA

**Keywords:** fast-breathing pneumonia, iCCM, sub-Saharan Africa, treatment failure, non-treatment

## Abstract

**Background:**

Despite recent progress, pneumonia remains the largest infectious killer of children globally. This paper describes outcomes of not treating community-diagnosed fast-breathing pneumonia on patient recovery.

**Methods:**

We conducted an exploratory subanalysis of an observational prospective cohort study in Malawi. We recruited children (2–59 months) diagnosed by community health workers with fast-breathing pneumonia using WHO integrated community case management (iCCM) guidelines. Children were followed at days 5 and 14 with a clinical assessment of recovery. We conducted bivariate and multivariable logistic regression for the association between treatment of fast-breathing pneumonia and recovery, adjusting for potential confounders.

**Results:**

We followed up 847 children, of whom 78 (9%) had not been given antibiotics (non-treatment). Non-treatment cases had higher baseline rates of diarrhoea, non-severe hypoxaemia and fever. Non-recovery (persistence *or* worsening of symptoms) was 13% and 23% at day 5 in those who did receive and those who did not receive co-trimoxazole. Non-recovery, when defined as worsening of symptoms *only*, at day 5 was 7% in treatment and 10% in non-treatment cases. For both definitions, combined co-trimoxazole and lumefantrine-artemether (LA) treatment trended towards protection (adjusted OR (aOR) 0.28; 95% CI 0.12 to 0.68/aOR 0.29; 95% CI 0.08 to 1.01).

**Conclusion:**

We found that children who did not receive co-trimoxazole treatment had worse clinical outcomes; malaria co-diagnosis and treatment also play a significant role in non-recovery. Further research into non-treatment of fast-breathing pneumonia, using a pragmatic approach with consideration for malaria co-diagnosis and HIV status is needed to guide refinement of community treatment algorithms in this region.

**Strengths and limitations of this study**

- This is an exploratory subanalysis of a prospective observational study, with data on concurrent diagnoses, treatments and outcomes collected in a rural routine care setting.
- Oral antibiotics and lumefantrine-artemether treatment were not randomised, and therefore the study is subject to bias, although we adjusted for confounders and clustering in the analysis.
- Pneumonia diagnosis and recovery was based on the WHO integrated community case management guidelines, enhanced with pulse oximetry.

## Background

Pneumonia is estimated to kill 0.9 million children each year, with the highest burden in Africa.^1^ ^2^ Reductions in pneumonia mortality have been seen over the last decade for significant bacterial causes of child morbidity and mortality, with widespread introductions of vaccines for *Haemophilus influenzae type B* and *Streptococcus pneumoniae*.^3^ ^4^ Increased access to these effective vaccines elevates the importance of understanding the role of non-bacterial illnesses that cause fast breathing, such as viral respiratory infections and non-respiratory illnesses like malaria, so that children with increased respiratory rates receive appropriate care.

The integrated community case management (iCCM) guidelines recommended by the WHO stratify pneumonia severity and subsequent treatment according to clinical symptoms.^5^ Non-severe cases (age-adjusted fast breathing without chest indrawing or danger signs) are currently recommended for treatment at home with oral antibiotics; however, antibiotics may not be necessary. American and European guidelines do not recommend antibiotics for bronchiolitis, a lower respiratory infection that can present with fast breathing and therefore mimic WHO iCCM pneumonia.^6^ In sub-Saharan African settings, multiple studies have reported the overlap of malaria and pneumonia diagnoses, resulting in overtreatment for both conditions.^7–9^

Treatment failure—the persistence of symptoms or clinical deterioration following antibiotic initiation—10can have many causes, including incorrect initial diagnosis (eg, malaria and not pneumonia), host comorbidities (eg, malnutrition), poor antibiotic adherence, and viral or antibiotic-resistant causative organisms.^11^ Published treatment failure rates for fast-breathing pneumonia range from 7% to 21%.^12–15^ Recent evidence from South Asia suggests that treating fast-breathing pneumonia with a placebo has equivalent recovery rates to the recommended course of oral antibiotics, reporting a treatment failure (or non-recovery) rate of 8% with or without antibiotics.^14^

Research is needed into the non-treatment of fast-breathing pneumonia in an African setting, where HIV and malnutrition (key risks for poor outcomes^16^ ^17^) rates are high, and access to care may be more limited. In Malawi community health workers (CHWs) dispense oral antibiotics in the community based on clinical assessments, and in this setting non-treatment refers to the lack of antibiotic dispensing by the CHW, not a care givers refusal of treatment. We aimed to take advantage of prospective observational data to describe outcomes for children diagnosed with fast-breathing pneumonia at the community level in Malawi who did not receive an antibiotic compared with those who received co-trimoxazole.

## Methods

This analysis is based on data collected as part of a prospective cohort study to predict treatment failure in children treated with oral co-trimoxazole for fast-breathing pneumonia in Malawi. Full methods of data collection and management have previously been published.^15^ Data were collected from September 2013 to June 2014 in subpopulations of two districts in the central region of Malawi (Mchinji and Lilongwe districts). The population catchments were rural and made up of predominantly subsistence farmers, covering ∼50 000 people, of which 17% were estimated to be children under the age of 5 years.^18^

### Data collection

Briefly, patients were recruited from community-level primary care clinics (village clinics) run by government employed CHWs called Health Surveillance Assistants. Information from the initial clinical assessment (including pulse oximetry with Lifebox) and diagnosis was recorded on case report forms by the CHW, along with their referral or treatment decision. For each CHW, local village-level data collectors (VDCs) conducted follow-up interviews in recruited patients' homes at days 5 and 14, with the day of diagnosis being day 0. VDCs attended village clinics to recruit patients and at follow-up visits conducted a clinical assessment of the child (including pulse oximetry), asked about additional care seeking and antibiotic adherence. Children identified as ‘not recovered’ at follow-up were referred by the VDC back to the CHW for further assessment and if needed, referral or treatment. We conducted *data quality assurance* exercises, including a vital signs standardisation audit conducted by a paediatric pulmonologist (EDM) and random Global Positioning System (GPS) spot checks by field supervisors of follow-up locations.

### Definitions

The *cohort* was defined as all children aged 2–59 months with CHW-diagnosed WHO fast-breathing pneumonia.

*Fast-breathing pneumonia* was defined according to the 2013 WHO iCCM guidelines,^19^ as the presence of cough and/or difficulty breathing and fast breathing (>50 bpm for infants 2–11 months; >40 bpm for children 12–59 months), in the absence of any danger signs or chest indrawing. Danger signs were recorded based on caregiver's report and clinical observation, and included vomiting everything, unable to feed, convulsions in the previous 24 hours, sleepy or unconscious, and signs of severe respiratory distress including grunting, severe hypoxaemia (peripheral oxygen saturation (SpO_2_) <90%), nasal flaring and head nodding. Wheeze was not specifically assessed or recorded. All children who were severely malnourished (mid-upper arm circumference (MUAC) <11.5 cm) were also referred. We defined moderate malnutrition as a MUAC between 11.5 and 13.5 cm, well nourished as >13.5 cm, and non-severe hypoxaemia as a SpO_2_ of 90–94%. Malaria diagnosis was based on clinical presentation and presumptive treatment of fever, without the use of rapid diagnostic tests or laboratory confirmation, as this was standard care at the time of this study. HIV testing was not offered by participating CHWs as this service also was not standard care.

Children were defined as ‘complete treatment’ or ‘non-treatment’ cases. *Complete treatment cases* were those who received and completed >80% of the 5-day, twice daily course of co-trimoxazole (where one dose is: 1/2 tablet for 2–11 months; 1 tablet for 12–59 months), according to standard guidelines in Malawi at the time of the study. *Non-treatment cases* were those who did not receive antibiotics from the CHW at their diagnosis or from any other source, according to caregiver's report and VDC inspection. *Non-adherent cases* were those who received antibiotics but completed <80% of the recommended doses. There were 80 cases (9%) that were non-adherent and we excluded these cases from the analysis to focus on non-treatment rather than incomplete or partial treatment effects.

*Non-recovery* definitions are presented in box 1. We used two definitions for non-recovery at day 5: (1) the persistence or worsening of symptoms, including fast breathing for age (definition 1—persistence and/or progression); (2) the worsening of symptoms, without considering fast breathing for age (definition 2—progression). A change in antibiotic was based on caregiver's report, with a named antibiotic prescribed by any level of clinical staff, and where possible visually verified by the VDC. At day 14, *relapse* (patients who had previously recovered) and *non-recovery* (classified as not recovered on day 5) were defined using the same parameters.

Box 1 **Non-recovery definitions**

- *Non-recovery definition 1 (persistence and/or progression)*—presence of any of the following on day 5 of follow-up: fast breathing for age or parameters from non-recovery definition 2.
- *Non-recovery definition 2 (progression)*—presence of any of the following on day 5 of follow-up: axillary temperature >37.5°C, lower chest indrawing, any danger sign (as defined in the Methods section), change of antibiotic, hospital admission or death.
- *Relapse/non-recovery—*presence of any of the following on day 14 of follow-up in children cured on day 5 (relapse) or not-recovered on day 5 (non-recovery): fast breathing for age or parameters from non-recovery definition 2.

### Analysis

All analyses were carried out using Stata SE13. We described the non-treatment and treatment cases. We conducted bivariate and multivariable analysis of outcomes at day 5 (using both definitions of non-recovery) for treatment and non-treatment cases; we did not do this for day 14 outcomes due to the small number of relapse/non-recovery cases. We considered the following variables in the multivariable analysis: clinical malaria diagnosis and lumefantrine-artemether (LA) treatment; other clinical diagnoses and treatments; initial clinical presentation including—temperature, SpO_2_, respiratory rate and malnutrition; age; and gender. These variables were included based on previously published associations with treatment failure,^15^ and differences observed between treatment and non-treatment groups to adjust for potential confounding. We imputed missing data values for those patients who completed follow-up at day 5, using chained equations with 10 rounds of imputation.^20^ A total of 226 records had an imputed value (24%); a sensitivity analysis of complete case analysis is available in online supplementary file 1. Robust SEs were used to account for clustering with the Stata command *-vce(robust)-*, and intracluster correlation coefficients reported. A bivariate analysis, stratified by presence of fever at CHW diagnosis is presented in online supplementary file 2.

10.1136/bmjopen-2016-011636.supp1 supplementary file 1

10.1136/bmjopen-2016-011636.supp2 supplementary file 2

### Ethics

Informed verbal consent for follow-up and clinical assessment was sought from the accompanying caregiver.

### Results

**Figure 1:**
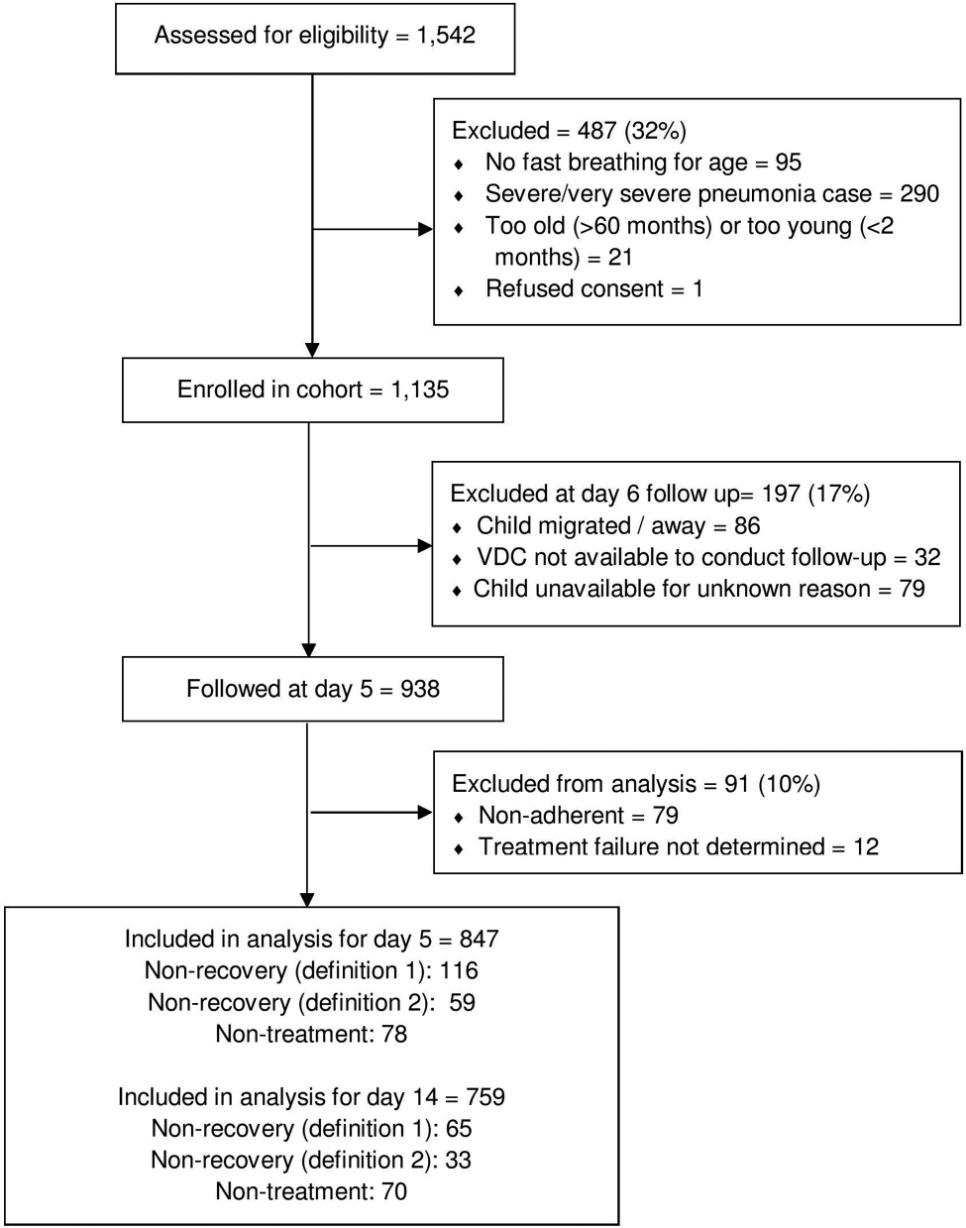
Case recruitment.

A total of 1542 cases were assessed for eligibility. Of these, 487 (32%) were not eligible (eg, too old or without fast breathing), 197 (17%) were lost to follow-up due to respondent unavailability, VDC unavailability or unknown (figure 1). Overall 938 cases were followed at day 5, with 847 cases retained for analysis, and of these 754 (89%) were also followed at day 14. Of these followed cases 78 (9%) were non-treatment cases.

**Figure 2:**
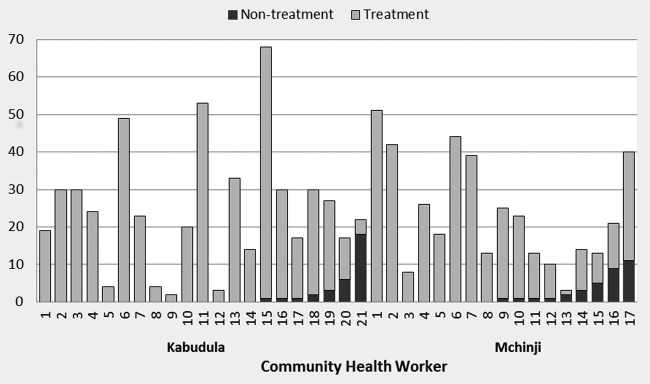
Distribution of case recruitment and non-treatment by community health worker.

Table 1 summarises baseline demographic and clinical signs of treatment and non-treatment cases. Treatment cases were similar to non-treatment cases in terms of demographics, but the cases that did not receive antibiotics had higher baseline reports of diarrhoea, additional treatments given and higher rates of non-severe hypoxaemia and fever. Figure 2 presents the proportion of non-treatment cases by CHW. Non-treatment cases were seen in 16 of the 38 CHW clusters and there was considerable variation in the number of children recruited and which received treatment between the CHWs. The intracluster correlation coefficients for non-recovery were 0.146 (95% CI 0.058 to 0.234) and 0.082 (95% CI 0.022 to 0.142) based on definitions 1 and 2, respectively.

**Table 1:** Description of demographics and clinical presentation in treatment and non-treatment cases

	Treatment (%) N=769	Non-treatment (%) N=78
*Variable*		
District		
Mchinji	350 (46%)	33 (42%)
Lilongwe	419 (54%)	45 (58%)
Age (months)		
2–11	238 (31%)	21 (27%)
12–23	252 (33%)	25 (32%)
24–59	279 (36%)	32 (41%)
Gender		
Girl	395 (51%)	33 (42%)
Boy	359 (47%)	43 (55%)
Missing	15 (2%)	2 (3%)
*Other treatments and diagnoses*		
Clinical malaria diagnosis	309 (40%)	25 (32%)
Diarrhoea diagnosis	22 (3%)	8 (10%)
Other diagnosis*	63 (8%)	5 (6%)
LA treatment given	386 (50%)	42 (54%)
Paracetamol given	383 (50%)	48 (61%)
Other treatment given**	15 (2%)	8 (10%)
Clinical features	Mean (SD) N (%)	Mean (SD) N (%)
MUAC (cm)	14.7 (1.4)	15.0 (1.6)
Moderate malnutrition	108 (14%)	9 (12%)
Missing	149 (19%)	17 (22%)
Temperature (°C)	37.1 (1.0)	37.4 (1.0)
Fever	252 (33%)	35 (45%)
Missing	34 (4%)	9 (12%)
Respiratory rate (bpm)		
2–11 months	55.5 (5.4)	56.0 (5.8)
12–59 months	47.6 (7.2)	49.3 (5.8)
Very fast	40 (5%)	3 (4%)
Missing	40 (5%)	3 (4%)
Oxygen saturation (SpO_2_%)	96.4 (1.9)	96.2 (2.7)
Non-severe hypoxaemia	117 (15%)	21 (27%)
Missing	11 (1%)	–

Very fast breathing: >70 bpm in 2–11 months old and >60 bpm in 12–59 months old. Moderate malnutrition: MUAC 11.5–13.5 cm. Non-severe hypoxaemia: SpO_2_90–94%.

*Other diagnoses includes: ear infection (n=2), rash (n=2) or other unspecified (n=64).

**Other treatments include salbutamol (n=5), aspirin (n=9) and any creams (n=9).

LA, lumefantrine-artemether; MUAC, mid-upper arm circumference; SpO_2_, oxygen saturation.

The rate of non-recovery (persistence and/or progression—definition 1) for non-co-trimoxazole treatment cases was 23% and for treatment cases was 13%. Children treated with both co-trimoxazole and LA had a non-recovery rate of 12% (unadjusted OR 0.32; 95% CI 0.15 to 0.70). Considering progression only (definition 2), non-recovery was 7% and 10% in those who did and did not receive antibiotics, respectively; non-recovery decreased to 5% in children with combined co-trimoxazole and LA treatment (unadjusted OR 0.44; 95% CI 0.14 to 1.36). Unadjusted results, including relapse/non-recovery at day 14 are presented in table 2. Notably, we did not register any deaths during the 2-week follow-up period.

**Table 2:** Association of treatment with co-trimoxazole and LA with non-recovery at days 5 and 14

	Day 5 N=116/847			Day 14 N=65/754		
	n/N (%)	OR	95% CI	n/N (%)	OR	95% CI
Definition 1						
No co-trimoxazole	18/78 (13%)	1.00		12/70 (8%)	1.00	
Co-trimoxazole	98/769 (23%)	0.49	0.28 to 0.86	53 684 (17%)	0.41	0.21 to 0.80
No treatment	11/36 (31%)	1.00		9/31 (29%)	1.00	
LA only	7/42 (17%)	0.45	0.15 to 1.34	3/39 (8%)	0.20	0.05 to 0.83
Co-trimoxazole only	50/383 (13%)	0.34	0.16 to 0.74	23/341 (7%)	0.18	0.07 to 0.43
Co-trimoxazole+LA	48/386 (12%)	0.32	0.15 to 0.70	30/343 (9%)	0.23	0.10 to 0.55
	N=59/847			N=33/754		
Definition 2						
No co-trimoxazole	8/78 (10%)	1.00		10/70 (15%)	1.00	
Co-trimoxazole	51/769 (7%)	0.62	0.28 to 1.36	23/684 (4%)	0.21	0.10 to 0.47
No treatment	4/36 (11%)	1.00		7/31 (24%)	1.00	
LA only	4/42 (10%)	0.84	0.19 to 3.64	3/39 (8%)	0.26	0.06 to 1.12
Co-trimoxazole only	31/383 (8%)	0.70	0.23 to 2.12	8/341 (2%)	0.08	0.03 to 0.24
Co-trimoxazole+LA	20/386 (5%)	0.44	0.14 to 1.36	15/343 (5%)	0.15	0.06 to 0.41

LA, lumefantrine-artemether.

Results of the bivariate and multivariate analysis are presented in table 3. For both definitions of non-recovery at day 5, having either clinically diagnosed malaria or any other diagnosis was associated with increased odds of a poor outcome. For definition 1, being older and having non-severe hypoxaemia also showed an increased risk for non-recovery. The complete case analysis demonstrated similar results to the imputed model, with the exception of co-trimoxazole only treatment for progression to more severe illness (definition 2), which trended towards recovery (adjusted OR (aOR) 2.02; 95% CI 0.29 to 14.01). However, the wide CIs demonstrate the uncertainty and lack of power in the complete case analysis (see online supplementary file 1).

**Table 3:** Bivariate and multivariable analysis following multiple imputation of non-recovery at day 5

	Unadjusted			Adjusted*		
Variable	OR	95% CI	p Value	OR	95% CI	p Value
*Treatment failure definition 1 (persistence and/or progression)*						
No treatment	1.00			1.00		
LA only	0.45	0.15 to 1.34	0.152	0.59	0.18 to 1.98	0.397
Co-trimoxazole only	0.34	0.16 to 0.74	0.006	0.57	0.25 to 1.32	0.187
Co-trimoxazole+LA	0.32	0.15 to 0.70	0.004	0.28	0.12 to 0.68	0.005
Clinical malaria diagnosis	1.58	1.07 to 2.35	0.022	2.21	1.30 to 3.75	0.003
Other diagnosis**	1.85	1.08 to 3.17	0.025	1.89	1.08 to 3.28	0.025
Other treatment***	1.51	1.01 to 2.26	0.042	1.63	0.99 to 2.68	0.052
Gender (male)	1.29	0.87 to 1.92	0.206	1.36	0.90 to 2.06	0.149
Age (months)						
2–11	1.00			1.00		
12–23	2.32	1.34 to 4.00	0.003	2.60	1.45 to 4.65	0.001
24–59	2.07	1.20 to 3.56	0.009	2.40	1.34 to 4.27	0.003
Moderate malnutrition	1.51	0.89 to 2.55	0.123	1.44	0.85 to 2.45	0.172
Fever	0.67	0.42 to 1.05	0.079	0.63	0.39 to 1.02	0.058
Very fast breathing	0.86	0.33 to 2.23	0.756	0.69	0.24 to 2.01	0.500
Non-severe hypoxaemia	1.92	1.20 to 3.06	0.006	1.75	1.05 to 2.93	0.032
*Treatment failure definition 2 (progression)*						
No treatment	1.00			1.00		
LA only	0.84	0.19 to 3.64	0.818	0.66	0.13 to 3.43	0.617
Co-trimoxazole only	0.70	0.23 to 2.12	0.534	0.89	0.27 to 2.90	0.840
Co-trimoxazole+LA	0.44	0.14 to 1.36	0.152	0.29	0.08 to 1.01	0.052
Clinical malaria diagnosis	1.77	1.04 to 3.01	0.035	3.24	1.59 to 6.60	0.001
Other diagnosis**	1.95	0.97 to 3.89	0.060	2.21	1.10 to 4.43	0.025
Other treatment***	1.01	0.59 to 1.71	0.984	1.23	0.62 to 2.42	0.552
Gender (male)	1.68	0.97 to 2.90	0.062	1.77	0.99 to 3.17	0.054
Age (months)						
2–11	1.00			1.00		
12–23	0.50	0.23 to 1.11	0.088	0.55	0.24 to 1.26	0.155
24–59	1.48	0.81 to 2.72	0.203	1.68	0.87 to 3.22	0.120
Moderate malnutrition	1.78	0.89 to 3.55	0.101	1.32	0.63 to 2.75	0.457
Fever	0.69	0.36 to 1.30	0.248	0.76	0.38 to 1.50	0.427
Very fast breathing	0.70	0.16 to 2.98	0.631	0.79	0.17 to 3.63	0.763
Non-severe hypoxaemia	1.38	0.71 to 2.69	0.340	1.39	0.69 to 2.77	0.358

Fever: temperature ≥37.5°C; mild hypoxaemia: oxygen saturation 90–94%.95%; moderate malnutrition: MUAC 11.5–13.5 cm; very fast breathing; >70 bpm in 2–11 months and >60 bpm in 12–59 months.

*The adjusted model included all the variables from the bivariate analysis.

**Other diagnoses include ear infection, rash or other unspecified.

***Other treatments include salbutamol, aspirin and any creams.

LA, lumefantrine-artemether.

Of the patients with malaria diagnosis 81% received LA, while 36% of those who received LA had no malaria diagnosis documented (correlation 0.49)—this incorrect treatment with LA was higher in non-treatment cases (40% vs 31%), as was correct treatment for concurrent malaria diagnosis (84% vs 81%). The association of co-trimoxazole and LA treatment on recovery for both definitions demonstrated protective effects; for definition 1 (persistence/progression) combined treatment showed a 72% decrease in non-recovery (aOR 0.28; 95% CI 0.12 to 0.68). In definition 2 (progression), the effect size was similar for combined treatment (aOR 0.29; 95% CI 0.08 to 1.01) although decreased power led to wider CIs.

## Discussion

This paper describes non-recovery in children diagnosed with fast-breathing pneumonia in the community in rural Malawi, specifically looking at the impact of co-trimoxazole and LA treatment. Our results suggest that oral co-trimoxazole treatment may be beneficial at the community level for children with fast-breathing pneumonia as part of routinely delivered community-based care. We found concurrent diagnoses, including malaria, to be important factors in non-recovery, as well as poorer guideline adherence by CHWs when treating children with possible baseline comorbidities, highlighting CHW inconsistencies when implementing iCCM guidelines. The role of malaria and treatment with both co-trimoxazole and LA in this setting needs further research and understanding to refine clinical diagnosis and treatment guidelines.

Interestingly, we found that the children who did not receive co-trimoxazole treatment had more comorbidities at baseline (more non-severe hypoxaemia, diarrhoea and fever), and received more alternative treatments such as salbutamol tablets, aspirin and skin creams. These baseline differences could explain the CHW decision to not administer oral antibiotics, if the CHW suspected them of having an alternative diagnosis to pneumonia (ie, malaria and/or diarrhoea with dehydration), despite meeting WHO iCCM criteria—reflecting the lack of specificity of fast breathing for pneumonia. Skin creams were likely dispensed to treat rashes, which could be due to a variety of causes such as self-limiting (eg, a viral exanthema) or higher risk infections (eg, *Staphylococcus aureus*), especially if the child is malnourished or HIV affected. While not recommended as part of iCCM care and not common in our study (n=5), a CHW may attempt to treat ‘noisy’ breathing in the community with salbutamol tablets. A child can have ‘noisy’ breathing due to many causes including a benign self-limiting viral upper respiratory tract infection, or diseases like bronchiolitis or croup that may lead to slower recovery, or non-recovery, as was observed in both definitions. None of these conditions would necessarily improve with salbutamol treatment, and all could result in a secondary bacterial infection that would benefit from a course of antibiotics. Alternatively the CHW may have diagnosed them with more severe illness and recommended them for referral, with this being misunderstood by the caregiver or not carried out, resulting in non-treatment and poor recovery.

This study has highlighted the variation in both numbers of reported pneumonia cases and treatment between different CHWs. We did not capture information on reasons for non-treatment; however, changes in funding for essential drug procurement in Malawi were occurring during the time of this study, and it is possible that this resulted in localised drug stock-outs and therefore non-treatment. Alternatively non-treatment and low case ascertainment may reflect poor quality of care and implementation of treatment guidelines by CHWs. The recent introduction of an mHealth drug stock system (cStock) into Malawi aims to address the issue of stock-outs, although an initial baseline survey found that 27% of CHWs did not have all essential drugs available.^21^ Even with systems such as cStock, close supervision and quality control in the use of CHWs for treatment of childhood illnesses is necessary to ensure consistent and accurate implementation of standardised treatment guidelines.

The multivariate analysis, for both definitions of non-recovery, demonstrated the association of empiric malaria co-diagnosis with non-recovery, and this was particularly pronounced when using progression of clinical symptoms as the definition of non-recovery. This association remained even once treatment with both co-trimoxazole and LA were considered—potentially a reflection of the poor correlation between the diagnosis and treatment, with almost 20% of malaria cases not receiving LA treatment. These relationships suggests that non-recovery at day 5 is being driven by either co-infection with malaria or initial misdiagnosis (and therefore mistreatment) of malaria as pneumonia. Co-trimoxazole has been shown to prevent malaria infections as part of prophylaxis treatment for HIV patients and to be an effective malaria treatment.^22^ Considering evidence from prior studies demonstrating considerable overlap between malaria and pneumonia, as defined by WHO clinical definitions^7–9^ co-trimoxazole may be treating both bacterial pneumonia and malaria infections in this population. Despite our findings suggesting that combined co-trimoxazole and LA treatment may be beneficial in children meeting the WHO iCCM fast-breathing pneumonia definition our data support the need for more refined treatment algorithms in malaria endemic regions.

The main limitation of this study was that it was not randomised. As a subanalysis of an observational cohort designed to investigate predictors of treatment failure, all children should have received oral co-trimoxazole for home treatment of fast-breathing pneumonia, according to iCCM guidelines. However, considering this was a quasi-programmatic setting, breaches in guideline implementation were seen among some of the CHW—demonstrating considerable inter-CHW variation. This analysis takes advantage of these failures to describe outcomes in non-treatment cases; however, this may reduce the generalisability of the results to other settings. We see that children were not randomly allocated to receive or not receive antibiotics (the same being true for LA treatment), therefore our analysis is subject to selection bias at the CHW level. Owing to the lack of blinding (both of the care giver and CHW) the apparent benefit among treated children could, in part, reflect a placebo effect for some caregiver-reported symptoms if they were pleased to have received treatment, especially if supplies were scarce. Similarly, as the CHWs were not blinded to treatment, their assessment of respiratory rate and other signs and symptoms may have been biased; and this could have been confounded by socioeconomic factors or knowledge of comorbidities such as HIV status. We had a 17% loss to follow-up which may lead to biases in the analysis. However, we have previously published that were only minimal differences seen in nutrition and PCV13 vaccination status between those lost and followed up, therefore it is unclear if this would have affected the results.^15^

We suggest that these results be cautiously interpreted as preliminary data and not conclusive on the impact of non-treatment of fast-breathing pneumonia in a high HIV, malaria endemic setting. As this was a secondary analysis without an a priori sample size, we are aware that our study lacked power to test equivalency; however, we still found a relationship between treatment and outcome. Further research, such as a carefully monitored randomised equivalency study, is needed in the sub-Saharan African setting which accounts for HIV status and laboratory-confirmed malaria co-diagnosis to build on this exploratory analysis. However, caution is needed not to use a study design method which is completely explanatory. As rural CHW settings may not see point-of-care diagnostic tools for malaria and HIV (let alone universal coverage), a study design which removes all of this ‘noise’ may lead to a false-positive finding of equivalence, while in the reality of suboptimal diagnosis and referral, antibiotics could still be beneficial. A balance between pragmatic and explanatory may be more appropriate, an approach which has been highlighted in the revised PRECIS-2 tool^23^—a validated research tool for delivering trials that are fit for purpose. In addition, an investigation into the use of placebos in a programmatic setting in South Asia is needed, to confirm the findings from the trial setting in practice.

Our results suggest that non-treatment of children meeting the current WHO iCCM definition for fast-breathing pneumonia would not be advisable in a rural sub-Saharan African setting with malaria endemicity. Malaria co-diagnosis and treatment play a significant role in non-recovery and so any trial in which placebos are given in a similar setting would need to be implemented under close clinical supervision to avoid adverse outcomes, as well as tease out the complex relationships between antibiotics and LA with recovery. However, we did not record any deaths and only two children who did not receive an antibiotic sought additional care within our 2 weeks of follow-up.
