# Supplementary material for: Non-treatment of children with community health worker-diagnosed fast-breathing pneumonia in rural Malawi: exploratory subanalysis of a prospective cohort study
Source: BMJ Open. 2016 Nov 16;6(11):e011636. doi: 10.1136/bmjopen-2016-011636 (PMC5128900; doi:10.1136/bmjopen-2016-011636)
Supplement: supplementary file 1 [file bmjopen-2016-011636supp_file1.pdf]

Web-appendix 1: Complete case sensitivity analysis

Web-appendix 1: Complete case multivariable analysis of non-recovery at day 5			
**Treatment Failure Definition 1 (persistence and/or progression) N = 85/620**			
**Variable**	**Odds ratio**	**95% CI**	**p-value**
No treatment	1.00		
LA only	0.44	0.10, 1.93	0.275
Cotrimoxazole only	0.61	0.22, 1.70	0.340
Cotrimoxazole + LA	0.28	0.10, 0.79	0.016
Clinical malaria diagnosis	1.63	0.89, 2.98	0.111
Other diagnosis*	1.29	0.63, 2.62	0.490
Other treatment**	1.54	0.83, 2.83	0.168
Gender (male)	1.31	0.81, 2.11	0.265
Age: 2-11 months	1.00		
12-23 months	2.55	1.24, 5.22	0.011
24-59 months	1.81	0.86, 3.79	0.116
Moderate malnutrition	1.48	0.80, 2.74	0.210
Fever	0.58	0.34, 1.01	0.053
Very fast breathing	1.19	0.37, 3.76	0.773
Non-severe hypoxemia	1.41	0.78, 2.56	0.257
**Treatment failure Definition 2 (progression) N = 38/620**			
No treatment	1.00		
LA only	0.45	0.02, 8.67	0.598
Cotrimoxazole only	2.02	0.29, 14.01	0.478
Cotrimoxazole + LA	0.51	0.07, 3.94	0.521
Clinical malaria diagnosis	3.08	1.26, 7.52	0.014
Other diagnosis	1.19	0.39, 3.65	0.764
Other treatment	1.13	0.46, 2.78	0.796
Gender (male)	1.84	0.89, 3.84	0.102
Age: 2-11 months	1.00		
12-23 months	0.57	0.21, 1.53	0.263
24-59 months	1.08	0.46, 2.52	0.861
Moderate malnutrition	1.32	0.57, 3.05	0.516
Fever	0.66	0.28, 1.58	0.355
Very fast breathing	0.83	0.11, 6.14	0.856
Non-severe hypoxemia	1.02	0.41, 2.56	0.963

*Other diagnoses includes: ear infection, rash or other unspecified.
**Other treatments include: salbutamol, aspirin and any creams.
LA: lumefantrine artemether; Moderate malnutrition: MUAC 11.5 – 13.5 cm; Non-severe hypoxemia: oxygen saturation 90 – 94%.95%; Fever: temperature ≥37.5°C; Very fast breathing: >70 breaths/minute in 2-11 months and >60 breaths/minute in 12-59 months; CI: 95% confidence interval
