# Supplementary material for: Non-treatment of children with community health worker-diagnosed fast-breathing pneumonia in rural Malawi: exploratory subanalysis of a prospective cohort study
Source: BMJ Open. 2016 Nov 16;6(11):e011636. doi: 10.1136/bmjopen-2016-011636 (PMC5128900; doi:10.1136/bmjopen-2016-011636)
Supplement: supplementary file 2 [file bmjopen-2016-011636supp_file2.pdf]

Web-appendix 2: Bivariate analysis, stratified by fever

**Web-appendix 2: Association of treatment with cotrimaxazole and LA with non-recovery at day 5 and day 14 – a stratified analysis according to fever at diagnosis**							
		**Day 5**			**Day 14**		
		**N (%)**	**OR**	**95% CI**	**N (%)**	**OR**	**95% CI**
**Definition 1**							
Fever	No treatment	3 (30%)	1.00		2 (20%)	1.00	
	LA only	2 (8%)	0.20	0.03, 1.47	1 (4%)	0.17	0.01, 2.19
	Cotrimoxazole only	10 (12%)	0.32	0.07, 1.42	5 (7%)	0.28	0.05, 1.70
	Cotrimoxazole + LA	14 (8%)	0.21	0.05, 0.91	14 (9%)	0.41	0.08, 2.10
No fever	No treatment	7 (32%)	1.00		7 (39%)	1.00	
	LA only	3 (25%)	0.71	0.15, 3.49	0	-	-
	Cotrimoxazole only	38 (13%)	0.32	0.12, 0.84	17 (7%)	0.11	0.04, 0.32
	Cotrimoxazole + LA	27 (14%)	0.34	0.13, 0.92	13 (7%)	0.12	0.04, 0.38
**Definition 2**							
Fever	No treatment	0	1.00		1 (11%)	1.00	
	LA only	0	-	-	1 (4%)	0.35	0.02, 6.23
	Cotrimoxazole only	10 (12%)	5.54	1.68, 18.24	3 (4%)	0.34	0.03, 3.65
	Cotrimoxazole + LA	4 (2%)	-	-	7 (5%)	0.41	0.04, 3.74
No fever	No treatment	3 (14%)	1.00		6 (35%)	1.00	
	LA only	2 (17%)	1.27	0.18, 8.87	0	-	-
	Cotrimoxazole only	19 (7%)	0.44	0.12, 1.63	5 (2%)	0.04	0.01, 0.14
	Cotrimoxazole + LA	12 (6%)	0.42	0.11, 1.60	8 (5%)	0.09	0.03, 0.30
